# Prospects for the clinical application of exosomal circular RNA in squamous cell carcinoma

**DOI:** 10.3389/fonc.2024.1430684

**Published:** 2024-06-12

**Authors:** Rongzhong Wang, Shiyan Wang, Hua Jiang, Yingmei Lan, Shaobin Yu

**Affiliations:** ^1^ Department of Pharmacy, West China Hospital, Sichuan University, Chengdu, China; ^2^ Department of Rehabilitation Medicine, West China Hospital, Sichuan University, Chengdu, China; ^3^ Key Laboratory of Rehabilitation Medicine in Sichuan Province, West China Hospital, Sichuan University, Chengdu, China; ^4^ Division of Nephrology, National Clinical Research Center for Geriatrics, Kidney Research Institute, West China Hospital of Sichuan University, Chengdu, China

**Keywords:** squamous cell carcinoma, exosomes, circRNA, biomarkers, noninvasive diagnosis

## Abstract

Squamous cell carcinoma (SCC) is a prevalent malignancy affecting multiple organs in the human body, including the oral cavity, esophagus, cervix, and skin. Given its significant incidence and mortality rates, researchers are actively seeking effective diagnostic and therapeutic strategies. In recent years, exosomes and their molecular cargo, particularly circular RNA (circRNA), have emerged as promising areas of investigation in SCC research. Exosomes are small vesicles released into the extracellular environment by cells that contain biomolecules that reflect the physiological state of the cell of origin. CircRNAs, known for their unique covalently closed loop structure and stability, have garnered special attention in oncology and are closely associated with tumorigenesis, progression, metastasis, and drug resistance. Interestingly, exosomal circRNAs have been identified as ideal biomarkers for noninvasive cancer diagnosis and prognosis assessment. This article reviews the progress in research on exosomal circRNAs, focusing on their expression patterns, functions, and potential applications as biomarkers in SCC, aiming to provide new insights and strategies for the diagnosis and treatment of SCC.

## 1 Introduction

Squamous cell carcinoma (SCC) is an aggressive malignancy originating from the squamous epithelium that affects a wide array of organs, such as the oral cavity, esophagus, cervix, and skin (1–3). The incidence and mortality rates of SCC, a common cancer type, are significant concerns. Notably, SCC accounts for approximately 60% of all malignancies in the orofacial region and accounts for approximately 90% of all esophageal cancer cases (4). Furthermore, it constitutes the majority of cervical and skin cancer diagnoses (5–8). These statistics not only highlight the global prevalence of SCC but also underscore its severe public health implications. Unfortunately, the therapeutic landscape for SCC is fraught with challenges, as most patients are diagnosed at advanced stages, leading to poor treatment outcomes, high recurrence rates, and dismal prognoses. Therefore, the identification of effective diagnostic and prognostic biomarkers is crucial for enhancing patient survival and quality of life. In this context, exosomes and their cargo molecules have attracted significant scientific interest, particularly in the development and progression of SCC.

Exosomes are extracellular vesicles approximately 30–150 nm in diameter that are secreted into the extracellular milieu through exocytosis (9). Originating from the endosomal compartment, they fuse with the plasma membrane to be released from the cell (10). Exosomes are rich in biomolecules, including specific proteins, mRNAs, microRNAs, and notably, circular RNAs (circRNAs), which have recently attracted increased amounts of attention (11). The presence of these molecules not only reflects the biological state of the parent cell but also modulates the behavior of the recipient cells, facilitating intercellular communication and influencing disease pathogenesis (12). CircRNAs, a class of noncoding RNAs characterized by their covalently closed loop structure, are more stable than linear RNAs (13). CircRNAs generated within exosomes by back-splicing are strongly associated with the onset and progression of various diseases, particularly in oncology, where aberrant circRNA expression has been linked to cancer initiation, progression, metastasis, and chemoresistance (14–16). Given their rich circRNA content, exosomes are considered ideal biomarkers for noninvasive cancer diagnostics and prognosis.

In summary, exosomes and their encapsulated circRNAs play a pivotal role in SCC research due to their critical functions in disease biology and their vast potential in clinical applications. An in-depth investigation of the biological properties of exosomes and circRNAs, as well as their expression and function in SCC, promises to open new avenues for early diagnosis, therapeutic decision-making, and prognosis assessment in SCC. This paper reviews the current state of exosomal circRNA research, focusing on its expression patterns, functions, and potential as a biomarker in SCC, with the aim of providing fresh perspectives and strategies for the diagnosis and treatment of SCC.

## 2 Biological functions of exosomal circRNAs

Extracellular vesicles originating from endocytosis are nanoscale cell-derived vesicles with diameters ranging from approximately 30 to 150 nm. Most cell types are capable of producing extracellular vesicles, which circulate in bodily fluids such as blood, urine, saliva, and breast milk (17). The contents of extracellular vesicles consist of various growth factors, proteins, lipids, nucleic acids, lncRNAs, and circRNAs. Consequently, extracellular vesicles play critical biological roles in cell interactions, affecting multiple cellular activities in both healthy and disease states. Increasing evidence has demonstrated the role of extracellular vesicles in mediating intercellular communication, the tumor microenvironment, immune system function, development and differentiation, cell signaling, and viral replication (18).

Circular RNAs were initially discovered in RNA viruses and are widely present as a diverse class of endogenous noncoding RNAs (19). Initially, considered an aberrant splicing byproduct of RNA, circRNA forms a covalently closed continuous loop structure after backsplicing of exons, introns or both. This closed loop structure of circRNA prevents degradation by RNA exonucleases or RNases, making it more stable than linear RNA and potentially serving as a disease biomarker. Over 80% of circRNAs overlap with protein-coding regions, suggesting that circRNAs may play important roles in diseases or serve as novel biomarkers. CircRNAs function in various key ways, negatively regulating miRNA expression, acting as miRNA sponges, regulating splicing and transcription, posttranscriptionally modulating gene expression, and potentially influencing cell growth and invasion processes in various cancers, including gastric, colorectal, and esophageal cancers (17).

**Figure 1:**
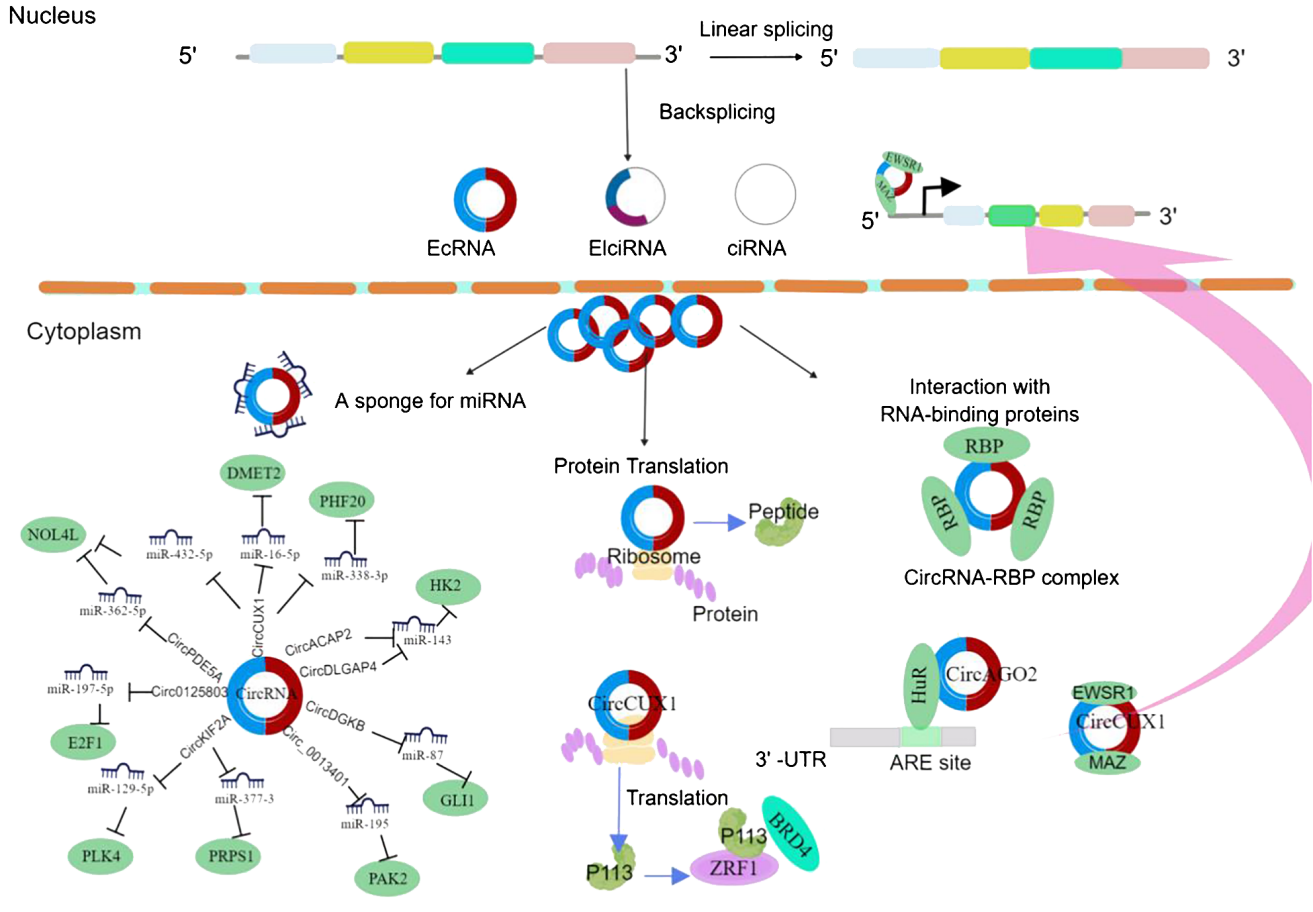
CircRNA biogenesis and mechanism in cancer cell progression. EcRNA, exonic circRNA; EIciRNA, exonic-intronic circRNA; CiRNA, intronic circRNA. EWSR1, EWS RNA-binding protein 1; MAZ, MYC-associated zinc finger protein; DMRT2, Doublesex and mab-3-related transcription factor 2; NOL4L, nucleolar protein 4-like; PHF20, PHD finger protein 20; BRD4, bromodomain protein 4; PLK4, polo-like kinase 4; PRPS1, phosphoribosyl pyrophosphate synthetase 1; GLI1, glioma-associated oncogene 1; AGO2, Argonaute 2; HK2, hexokinase 2; PAK2, p21-activated kinase 2; HuR, human antigen R.

Numerous studies have demonstrated that circRNAs have significant impacts on cancer progression and treatment (**Figure 1**), exerting regulatory effects on the tumor microenvironment and serving as potential therapeutic targets and novel cancer biomarkers. Research has shown that circRNAs are highly enriched in extracellular vesicles and can exist stably within them, with a significantly increased abundance of circRNAs compared to those secreted by normal cells (12, 20). Several circRNA species with potential biological functions have been identified in extracellular vesicles, especially in human serum-derived extracellular vesicles containing more than 1,000 circRNAs, possibly originating from tumors (21, 22). Some circRNAs have been detected in serum, urine, and tumor-derived extracellular vesicles. Tumor-derived extracellular vesicle circRNAs may participate in processes such as cell growth, angiogenesis, and epithelial–mesenchymal transition (23).

Studies have shown that the abundance of circRNA in extracellular vesicles is twice that of the parent cells and six times that of linear RNA (20). The sorting of circRNA into extracellular vesicles is regulated by changes in related miRNA levels in parent cells and facilitates the transfer of biological activity to recipient cells, thereby participating in intercellular communication (20). The nanoscale size and lipid bilayer structure of extracellular vesicles can prolong the circulation time of circRNAs and enhance their biological activity. Therefore, extracellular vesicle circRNAs possess both the targeting properties of extracellular vesicle-like transfer and the inherent biological functions of circRNAs, offering more significant regulatory advantages (24). Multiple studies have shown that tumor-specific circRNAs can be selectively packaged, secreted, and transported via tumor-derived extracellular vesicles, participating in the regulation of the tumor microenvironment to promote or inhibit tumor cell growth and metastasis. Extracellular vesicle circRNAs exhibit greater diagnostic sensitivity and specificity than freely circulating circRNAs in body fluids and could serve as valuable biomarkers for clinical diagnosis and prognosis (25).

## 3 The role of exosomal circRNAs in the diagnosis and progression of squamous cell carcinoma

CircRNAs play crucial roles in exosomes and are closely associated with the development of squamous cell carcinoma (26). A study revealed that high expression of circ_0000199 in oral squamous cell carcinoma is linked to increased recurrence and mortality rates, with its overexpression promoting tumor cell growth while its silencing exhibiting inhibitory effects (27). Similarly, circ_0072387 inhibits the progression of oral squamous cell carcinoma by regulating the expression of microRNA-503–5p (miR-503–5p), suggesting its potential as a therapeutic target for this disease (28). Research by Tang et al. further revealed that the high expression of circFNDC3B in serum exosomes is directly associated with the occurrence and development of esophageal squamous cell carcinoma, possibly serving as an independent risk predictor for aiding in early diagnosis (29). CircfNDC3B not only affects tumor cells through exosomal transfer but also functions through the miR-490–5p/TXNRD1 axis, further driving the progression of esophageal squamous cell carcinoma (30). Transfection of exosomal circ-CYP24A1 can inhibit the malignant behavior of cutaneous squamous cell carcinoma, offering new possibilities for noninvasive therapeutic strategies and potentially serving as a diagnostic marker (31). In CC, the expression level of circRNA_0000285 in cancer tissue samples was significantly greater than that in corresponding normal tissue samples. After knocking out circRNA_0000285, the expression of downstream FUS was significantly downregulated, which indicates that circRNA_0000285 may enhance the proliferation of cervical squamous cell carcinoma cells by upregulating FUS and provides potential therapeutic targets for research on cervical cancer (32). Additionally, a significant reduction in the impact of circ_0005576 silencing on cervical squamous cell carcinoma cells can be achieved by inhibiting miR-153–3p, confirming that circ_0005576 promotes cancer progression through the miR-153–3p/KIF20A axis and that its overexpression enhances cell proliferation and migration through a sponge effect on miR-153–3p (33). In conclusion, exosomal circRNAs not only serve as biomarkers for squamous cell carcinoma but also reveal potential therapeutic targets through their dynamic regulation. Future research should further explore the specific functions of these circRNAs and their potential applications in cancer prevention and treatment.

## 4 The role of exosomal circRNAs in squamous cell carcinoma metastasis

The development and metastasis of tumors represent a complex and multifaceted process that involves the synergistic action of multiple oncogenes and pathways. Recent studies have highlighted the indispensable role of circRNAs in this process. For instance, Li et al. demonstrated the upregulation of circMYOF in laryngeal cancer, where its silencing inhibited tumor cell growth, metastasis, and glycolysis, thereby suppressing overall tumor progression (34). Notably, circMYOF is overexpressed in the serum exosomes of patients with laryngeal cancer and promotes cell migration by regulating the miR-145–5p/OTX1 axis, thereby accelerating the progression of laryngeal cancer. In the case of esophageal squamous cell carcinoma, lymph node metastasis represents a critical stage of progression. Studies have shown that the level of circ_0026611 in serum exosomes is significantly elevated in patients with lymph node metastasis, and its expression level can serve as a biomarker for assessing lymph node metastasis (35). Exosomal circ_0026611 derived from ESCC cells can inhibit NAA10-mediated PROX1 protein acetylation and ubiquitination to increase the expression level of the PROX1 protein (36). These findings suggest that circ_0026611 has potential value in the clinical monitoring and prognostic assessment of esophageal squamous cell carcinoma (35). Exosomal circRNAs not only directly enhance the invasiveness of tumor cells but also influence the invasive behavior of cancer cells by regulating the tumor microenvironment (37). Particularly in the malignant progression of lymph node metastasis, the preoperative detection of circRNAs in serum exosomes could provide valuable information for the early identification of lymph node metastasis. Therefore, the analysis of exosomal circRNAs is highly important for understanding the pathways involved in squamous cell carcinoma metastasis and optimizing personalized treatment strategies.

## 5 The role of exosomal circRNAs in the treatment of squamous cell carcinoma

The significance of exosomal circRNAs in cancer biology is increasingly evident, as their potential as therapeutic targets and biomarkers has attracted increased amounts of attention (38, 39). Studies have revealed that exosomal circGDI2 regulates the malignant behaviors of oral squamous cell carcinoma cells by targeting the miR-424–5p/SCAI axis. CircRNA GDP dissociation inhibitor 2 (circGDI2) can be transferred via exosomes, positioning it as a novel exosome-based biomarker and therapeutic agent for treating oral squamous cell carcinoma (40). Cancer stemness and immune evasion are closely linked and play pivotal roles in tumor development and resistance to immunotherapy. Elevated circFAT1 promotes the activation of STAT3, thereby mediating a positive correlation between cancer stemness and immune evasion (41). CircFAT1 knockdown (KD) enhances CD8+ cell infiltration into the tumor microenvironment and augments the efficacy of PD-1 blockade immunotherapy. CircFAT1 interacts with cytoplasmic STAT3 to prevent SHP1-induced dephosphorylation of STAT3, promoting its activation and inhibiting STAT1-mediated transcription, establishing circFAT1 as a crucial regulator of cancer stemness and antitumor immunity. Similarly, overexpression of circ_0060927 reduces the activity and promotes the apoptosis of skin squamous cell carcinoma cells while inhibiting epithelial–mesenchymal transition (EMT) processes within cells. The overexpression of circ_0060927 impedes the Wnt signaling pathway via miR-4438, thereby affecting proliferation, apoptosis, and EMT in skin squamous cell carcinoma cells. Recent studies have suggested that exosomes can regulate the immune system and serve as potential agents in immune therapy. Exosomal circRNAs are increasingly regarded as a novel subset of endogenous RNAs that regulate target genes either by modulating miRNA activity or by forming complexes with target proteins. Studies indicate that exosomal circRNAs can affect the drug resistance of tumor cells, and their significant role as mediators of chemotherapy resistance is gradually being elucidated (42).

## 6 The role of circRNAs in the regulation of tumor chemotherapy and radiotherapy resistance

In the management of locally advanced malignancies, radiotherapy is frequently employed, either as an adjunct to surgical interventions or synergistically with chemotherapeutic agents (43). A significant barrier to the efficacy of these therapies in patients with advanced cancer is the development of resistance to chemotherapy (44). One of the pivotal mechanisms underpinning this resistance involves ATP-binding cassette B1 (ABCB1), a protein associated with multidrug resistance that is overexpressed in resistant cell lines and facilitates the extracellular expulsion of intracellular chemotherapeutic agents (45, 46). Consequently, targeting and inhibiting the expression of ABCB1 is a promising strategy for mitigating drug resistance in tumors (47). A notable example of this mechanism is observed in oral squamous cell carcinoma (OSCC), where circ_0109291 has been shown to enhance resistance to cisplatin by modulating ABCB1 expression primarily through the adsorption of miR-188–3p. This interaction provides fundamental insight for strategies aimed at reducing the prevalence of resistance in OSCC (48).

Furthermore, heat shock protein 27 (HSP27, also known as HSPB1) is a member of the small heat shock protein superfamily with Phospho-HSP27 (Ser15), Phospho-HSP27 (Ser78), and Phospho-HSP27 (Ser82) receptor sites (49). The function of HSP27 is regulated by posttranslational phosphorylation (50). In tongue squamous cell carcinoma (SCCT), HSP27 augments multidrug resistance by activating the NF-κB pathway (51). The overexpression of HSP27 is associated with cellular resistance to various chemotherapeutic agents, including cisplatin and staurosporin, in laryngeal squamous cell carcinoma (LSCC) through mechanisms involving the induction of cell cycle arrest and alterations in actin polymerization, which affect drug uptake (52). Interestingly, the circRNA circGNG7 has been shown to interfere with the phosphorylation of HSP27 at Ser78 and Ser82 in head and neck squamous cell carcinoma (HNSCC), thus impeding its phosphorylation within malignant signaling pathways and potentially reducing chemoresistance in HNSCC (53). In addition, circ-PKD2 in OSCC functions as a tumor suppressor by promoting the expression of autophagy-related 13 (Atg13) through the adsorption of miR-646, thereby enhancing sensitivity to cisplatin (54). Radiotherapy tolerability also serves as a critical prognostic indicator in patients with HNSCC. The upregulation of circCUX1 in radiation-resistant hypopharyngeal squamous cell carcinoma (HPSCC) is correlated with poorer survival outcomes (55). CircCUX1 interacts with caspase-1 mRNA, inhibiting its expression, which in turn modulates the inflammatory response of tumor cells to radiation, thereby fostering tolerance to radiation therapy.

## 7 Challenges and prospects

Exosomal circRNAs represent a cutting-edge area of research in oncology. These molecules are known to regulate key cancer cell processes, such as proliferation, invasion, migration, and metastasis, and hold potential as biomarkers for early cancer diagnosis. Increasing evidence has highlighted the significant role of exosomal circRNAs in the initiation and progression of squamous cell carcinoma (**Table 1**) (87). CircRNAs can modulate the expression of downstream oncogenic molecules through the circRNA-miRNA−mRNA axis, playing a crucial role in the progression of squamous cell carcinoma (17). The levels of specific exosomal circRNAs, which are correlated with tumor development in specific patients, have demonstrated potential as early diagnostic and prognostic biomarkers. These circRNAs circulate in bodily fluids via exosomes, enabling noninvasive detection. Additionally, exosomal circRNAs can influence the tumor microenvironment by facilitating communication between tumor cells and surrounding cells (such as immune and endothelial cells), regulating cancer cells’ ability to evade the immune system, and affecting angiogenesis (88). Exploring these mechanisms could provide insights for the development of new anticancer strategies. Drug resistance remains a critical factor in the failure of therapies for the treatment of squamous cell carcinoma. Some exosomal circRNAs have been shown to induce resistance in cancer cells to chemotherapy or radiotherapy by modulating intracellular signaling pathways (89). Therefore, the study of these circular RNAs not only aids in understanding the resistance mechanism but also may provide new approaches for overcoming this resistance.

**Table 1:** Mechanisms of action and biological functions of key circRNAs involved in SCC.

Cancer type	CircRNAs	Chromosome	Gene symbol	Splicing type	Expression	Targets/effectors	Biological function	Potential function	RF
OSCC	hsa_circ_0005379	chr10	GDI2	EIciRNAs	down	EGFR	Oncogenic functions	Therapeutic target	(56–58)
	circUHRF1	chr19	UHRF1	EIciRNAs	up	miR-526b-5p/c-Myc	Promotes proliferation	Therapeutic target	(59, 60)
	circCDR1as	chrX	CDR1	EIciRNAs	up	miR-671–5p	Promoted autophagy	Therapeutic target	(61)
	circANTRL1	chr10	ANTRL1	EIciRNAs	down	miR-23a-3p/PTEN	Induce cell cycle arrest	Therapeutic target	(62)
	circ_0000745	chr17	SPECC1	EIciRNAs	up	miR-488/CCND1	Induced cell cycle arrest	–	(57, 58, 63)
	has_circ_0055538	chr2	RMND5A	EIciRNAs	down	p53/Bcl-2/Caspase	Tumor suppressor	Therapeutic target	(64)
	circ_0005320	chr17	SEPT9	EIciRNAs	up	miR-486–3p/miR-637	Oncogenic functions	Therapeutic target	(65)
	hsa_circ_0001766	chr7	PDIA4	EIciRNAs	up	miR-877–3p/VEGFA	Oncogenic functions	Biomarker	(57, 58, 62)
	circEPSTI1	chr13	EPSTI1	EIciRNAs	up	miR-942–5p	Promote EMT, Oncogenic functions	Therapeutic target	(66, 67)
	circ_0000140	chr1	KIAA0907	EIciRNAs	down	miR-31, LATS2	Tumor suppressor	Therapeutic target	(68)
	circ_0109291	chr19	ZNF714	EIciRNAs	up	miR-188–3p	Promotes cisplatin resistance	–	(48, 57, 58)
	circ-PKD2	chr4	PKD2	EIciRNAs	down	miR-646, Atg13	Increase cisplatin sensitivity	Prognosis	(54)
LSCC	circZNF609	chr15	ZNF609	EIciRNAs	up	miRNA-134–5P/EGFR	Promotes proliferation	Therapeutic target	(69, 70)
	circCDR1as	chrX	CDR1	EIciRNAs	up	miR-7	Promotes proliferation	Prognosis and biomarker	(71, 72)
	hsa_circ_0006232	chr6	TRERF1	EIciRNAs	up	PTEN	Oncogenic functions	Therapeutic target	(57, 58, 73)
	circMYLK	chr3	MYLK	EIciRNAs	up	miR-195/cyclin D1	Promotes proliferation accelerates cell cycle transition	Therapeutic target	(74, 75)
	circ-CCND1	chr11	CCND1	EIciRNAs	up	miR-646/CCND1	Accelerates cell cycle transition	Therapeutic target	(57, 58, 76)
	circ_0000218	–	–	–	up	miR-139–3p/Smad3 axis	Oncogenic functions	Therapeutic target	(77)
	circPARD3	chr10	PARD3	EIciRNAs	up	PRKCI-Akt-mTOR	Inhibit autophagy	Therapeutic target and biomarker	(78)
	circCORO1C	chr12	CORO1C	EIciRNAs	up	let-7c-5p	Oncogenic functions	Therapeutic target	(79)
	hg19_circ_0005033	–	–	–	up	miR-4521	Oncogenic functions	Therapeutic target and biomarker	(80)
NPC	circ-NOTCH1	chr9	NOTCH1	EIciRNAs	up	miR-34c-5p/c-Myc	Oncogenic functions	–	(81)
	circCAMSAP1	chr9	CAMSAP1	EIciRNAs	up	SERPINH1/c-Myc	Oncogenic functions	Therapeutic target	(82, 83)
	circRPMS1	–	RPMS1	–	up	miR-203, miR-31, and miR-451	Promote EMT, Oncogenic functions	Therapeutic target	(84)
	circCRIM1	chr2	CRIM1	EIciRNAs	up	–	Promoted metastasis and EMT	Prognosis	(84, 85)
	circBART2.2	–	–	–	up	IRF3	Promotes immune escape	Therapeutic target	(86)
HPSCC	circGNG7	chr19	GNG7	EIciRNAs	down	Ser78, Ser82	Tumor suppressor	Prognostic and therapeutic target	(53)
	circCUX1	chr7	CUX1	EIciRNAs	up	caspase 1	Radiotherapy tolerance	Therapeutic target	(55)
SCC	circFAT1	chr4	FAT1	EIciRNAs	up	STAT3	Immunosuppressive environment	Therapeutic target	(41)

While exosomal circRNAs hold great potential as cancer biomarkers, their detection and identification still face technological challenges, particularly in terms of sensitivity and specificity in low-abundance samples (90). Moreover, the overlap of circular structures and sequences with their linear mRNA counterparts makes accurate assessment of circular RNA expression and function challenging. The mechanisms by which exosomal circRNAs contribute to cancer development are extremely complex and involve not only gene regulation and protein expression and function but also potentially unknown pathways, necessitating extensive and in-depth studies from the molecular to the systems biology level. Furthermore, the mechanisms by which circRNAs are enriched during the formation of exosomes remain unclear. Although exosomal circRNAs show potential in cancer diagnosis and therapy, their translation from basic research to clinical application faces multiple challenges, including but not limited to the validation of biomarkers, confirmation of therapeutic targets, and assessment of the safety and efficacy of corresponding treatment strategies.

## 8 Conclusion

Exosomal circRNAs have emerged as promising biomarkers and therapeutic targets in the study of squamous cell carcinoma. These molecules are integral to the onset, progression, and therapeutic response of tumors, providing innovative avenues for cancer diagnosis, prognostic assessment, and the formulation of new therapeutic approaches. Nonetheless, significant challenges persist, encompassing technological limitations, lack of comprehensive insight into their biological mechanisms, and barriers to clinical implementation. Future research needs to address these issues to fully harness the potential of exosomal circRNAs in the treatment of squamous cell carcinoma.

## Author contributions

RW: Writing – original draft. SW: Writing – review & editing. HJ: Writing – review & editing. YL: Supervision, Writing – review & editing. SY: Project administration, Writing – review & editing, Funding acquisition.
